# Application of alternative de novo motif recognition models for analysis of structural heterogeneity of transcription factor binding sites: a case study of FOXA2 binding sites

**DOI:** 10.18699/VJ21.002

**Published:** 2021-02

**Authors:** A.V. Tsukanov, V.G. Levitsky, T.I. Merkulova

**Affiliations:** Institute of Cytology and Genetics of Siberian Branch of the Russian Academy of Sciences, Novosibirsk, Russia; Institute of Cytology and Genetics of Siberian Branch of the Russian Academy of Sciences, Novosibirsk, Russia Novosibirsk State University, Novosibirsk, Russia; Institute of Cytology and Genetics of Siberian Branch of the Russian Academy of Sciences, Novosibirsk, Russia Novosibirsk State University, Novosibirsk, Russia

**Keywords:** transcription factor binding sites (TFBS), TFBS de novo searching, ChIP-seq, heterogeneity of TFBS, сайты связывания транскрипционных факторов (ССТФ), de novo поиск ССТФ, СhIP-seq, гетерогенность ССТФ

## Abstract

The most popular model for the search of ChIP-seq data for transcription factor binding sites (TFBS) is the positional weight matrix (PWM). However, this model does not take into account dependencies between nucleotide occurrences in different site positions. Currently, two recently proposed models, BaMM and InMoDe, can do as much. However, application of these models was usually limited only to comparing their recognition accuracies with that of PWMs, while none of the analyses of the co-prediction and relative positioning of hits of different models in peaks has yet been performed. To close this gap, we propose the pipeline called MultiDeNA. This pipeline includes stages of model training, assessing their recognition accuracy, scanning ChIP-seq peaks and their classification based on scan results. We applied our pipeline to 22 ChIP-seq datasets of TF FOXA2 and considered PWM, dinucleotide PWM (diPWM), BaMM and InMoDe models. The combination of these four models allowed a significant increase in the fraction of recognized peaks compared to that for the sole PWM model: the increase was 26.3 %. The BaMM model provided the main contribution to the recognition of sites. Although the major fraction of predicted peaks contained TFBS of different models with coincided positions, the medians of the fraction of peaks containing the predictions of sole models were 1.08, 0.49, 4.15 and 1.73 % for PWM, diPWM, BaMM and InMoDe, respectively. Thus, FOXA2 BSs were not fully described by only a sole model, which indicates theirs heterogeneity. We assume that the BaMM model is the most successful in describing the structure of the FOXA2 BS in ChIP-seq datasets under study.

## Abstract (ru)

. В настоящее время самой распространенной моделью поиска сайтов связывания транскрипционных факторов (ССТФ) в пиках ChIP-seq является позиционная весовая матрица (position weight matrix, PWM). Но эта модель не учитывает взаимосвязи между частотами встреч нуклеотидов в разных позициях ССТФ, поэтому не способна гарантировать определение всех возможных структурных вариантов ССТФ. На сегодняшний день уже предложены альтернативные модели, например BaMM и InMoDe, которые учитывают такие взаимосвязи. Однако применение этих моделей обычно сводилось к сравнению их точности с точностью традиционной модели PWM, тогда как анализ совместной встречаемости и относительного расположения ССТФ разных моделей в пиках не производился. В нашей работе мы предлагаем конвейер программ MultiDeNA, позволяющий сочетать разные модели de novo поиска ССТФ для выявления структурной гетерогенности ССТФ в данных ChIP-seq. Разработанный конвейер включает этапы построения моделей на основе заданного набора пиков, оценки точности распознавания моделей с помощью перекрестных тестов, выбора порогов, сканирования пиков ChIP-seq и классификацию пиков по результатам сканирования. С применением конвейера нами проведен анализ 22 экспериментов ChIP-seq для ТФ FOXA2 с помощью четырех моделей: PWM, diPWM, BaMM и InMoDe. Показано, что сочетание моделей позволяет существенно увеличить общее количество распознанных пиков (на 26.3 %) по сравнению с применением только PWM; при этом основной вклад в распознавание внесла модель BaMM. В значительной доле пиков разные модели распознают совпадающие ССТФ; однако для моделей PWM, diPWM, BaMM и InMoDe медианы доли пиков, которые содержали ССТФ только одной модели, составили 1.08, 0.49, 4.15 и 1.73 % соответственно. Таким образом, совокупность ССТФ FOXA2 не описывается полностью только одной моделью, что свидетельствует о наличии структурной гетерогенности в ССТФ у FOXA2.

## Introduction

Transcription factors (TFs) are proteins that can recognize certain regions of genomic DNA (TF binding sites, TFBS) (Lambert et al., 2018). The main function of TFs is to increase or decrease a level of gene transcription (Latchman, 2001). The key stage of the regulation of gene expression is TF binding to DNA. This binding initiates a chain of molecular events that ensure the assembly and regulate the activity of the pre-initiation complex of RNA polymerase II, both through direct or indirect contacts with the components of this complex, and through the involvement of various modifying chromatin and remodeling proteins. As a consequence, local changes in the structure of chromatin allow the transcription initiation (IwafuchiDoi, 2019; Srivastava, Mahony, 2020). Therefore, one of the most important tasks of modern molecular biology is to identify genomic TFBSs.

Currently, the ChIP-seq technique is widely used to solve this problem (Farnham, 2009; Park, 2009). This technique is based on the chromatin immunoprecipitation with antibodies to an investigated TF with consequent high-throughput sequencing of precipitated DNA. Primary ChIP-seq data processing identifies DNA regions, or peaks, in which a target TF was directly or through intermediate proteins binds DNA (Furey, 2012). However, lengths of peaks are usually equal to hundreds of bp, but a length of TFBS does not exceed 20–25 bp (Levitsky et al., 2007; Kulakovskiy et al., 2018). Thus, the next stage of the bioinformatics processing of ChIP-seq data is to search exact positions of TFBS in peaks. To date, many tools have been developed to solve this issue, the overwhelming majority of them are based on the model of position weight matrix (PWM) (Stormo, 2000), including such popular ones as ChIPMunk (Kulakovskiy, Makeev, 2009) and Homer (Heinz et al., 2010). It is no exaggeration to say that the use of different implementations of the PWM model are included in almost every pipeline of ChIP-seq data processing (Lloyd, Bao, 2019).

The application of the standard PWM-based approach to the processing of ChIP-seq data showed that for most TFs about a half of peaks did not contain detected PWM hits (Worsley Hunt, Wasserman, 2014; Gheorghe et al., 2019). Traditionally, this was associated with the main disadvantage of PWM, the hypothesis of independence of nucleotides frequencies in different positions of TFBS, which is not always true. This may negatively affect the recognition accuracy (Benos et al., 2002; Keilwagen, Grau, 2015). Therefore, alternative models of TFBS recognition have being developed. They took into account dependencies between nucleotides occurrences in a site model (Mathelier, Wasserman, 2013; Yang et al., 2014; Siebert, Söding, 2016; Eggeling et al., 2017; Gheorghe et al., 2019). Thus, the simplest alternative model was the dinucleotide position weight matrix (diPWM), it took into account dependences between adjacent nucleotides (Zhang M., Marr, 1993; Kulakovskiy et al., 2013). On the other hand, models such as BaMM (Siebert, Söding, 2016) and InMoDe (Eggeling et al., 2017) have been proposed. They were constructed using Markov chains, which took into account dependences of positions using the concept of Markov chain order, i. e. a length for which nucleotide frequencies can be mutually dependent (an order usually does not exceed 5 nt).

Authors of these alternative models proved that their models might outperform in recognition accuracy the standard PWM. However, these models were not applied to solve the problem of incomplete recognition of TFTS in ChIP-seq peaks. We assume that this problem is partially related to the structural heterogeneity of binding sites of TFs, and the number of recognized peaks can be significantly increased with the combination of different models together. In this case, the ChIP-seq peaks contain both predicted TFBS with application of a sole model, or with two models, etc. (Ignatieva et al., 2004; Levitsky et al., 2014, 2016). Earlier, we used the training sample of 53 known TF sites of the FOXA subfamily and analyzed ChIP-seq data of FOXA2 (Wederell et al., 2008; Wallerman et al., 2009) with alternative models ChIPMunk (PWM) (Kulakovskiy, Makeev, 2009) and SiteGA (Levitsky et al., 2007) with experimentally fitted model’s thresholds (EMSA experiment, electrophoretic mobility shift assay, shift in electrophoretic mobility analysis). We showed that both models together found FOXA2 sites in more than 95 % of peaks (Levitsky et al., 2014). This conclusion was consistent with the absence in literature any data about indirect interaction of this well-studied TF with genomic DNA.

The given example indicates that combination of alternative models with PWM model for analyzing ChIP-seq data is promising. However, until now there has been no systematic research on this topic. Alternative models of TFBS search are not widely used, despite that about 20 years ago it was proved that there is a dependence of the nucleotide frequencies in different positions in TFBS (Bulyk et al., 2002). As the indicator of the popularity of different models, we use the number of citations of papers devoted to specific de novo TFBS searching programs for ChIP-seq data analysis. Thus, at the end of 2020, papers devoted to the implementation of the PWM model MEME, HOMER and ChIPMunk (Bailey, Elkan, 1994; Heinz et al., 2010; Kulakovskiy et al., 2010; Machanick, Bailey, 2011) have the total number of citations over 6000. However, papers devoted to alternative models BaMM, InMoDe and diChIPMunk (Kulakovskiy et al., 2013; Siebert, Söding, 2016; Eggeling et al., 2017; Kiesel et al., 2018) have about 50 citations. Moreover, specific studies of individual ChIP-seq experiments were usually analyzed only with the standard PWM model. This situation we explain as follow. First, the PWM model is understandable and anyone can simply interpret it. Second, advantages of alternative models are insufficiently understandable. E. g., hardly anyone thought that alternative models were able systematically to find out TFBS of a different structure

In this paper, we propose the pipeline that combines four de novo models of TFBS search, namely ChIPMunk/ diChIPMunk implementations of PWM/diPWM (Kulakovskiy et al., 2010, 2013), and the Markov models InMoDe (Eggeling et al., 2017) and BaMM (Siebert, Söding, 2016). The pipeline evaluates the recognition accuracy of these models, selects their thresholds and classifies ChIP-seq peaks by comparing respective scan results. This approach expands the understanding of TFBS structural diversity, especially in cases when the PWM model is unable to find TFBS in a peak. We applied the pipeline for 22 ChIP-seq datasets for TF FOXA2.

## Materials and methods

For the analysis we used the set of preprocessed 22 ChIPseq datasets for TF FOXA2 in the bed format from the ReMap database http://remap.univ-amu.fr/ (Chèneby et al., 2020), see the Table. Only the best 4000 peaks we used for analysis in each sample (see below).

**Table 1.**
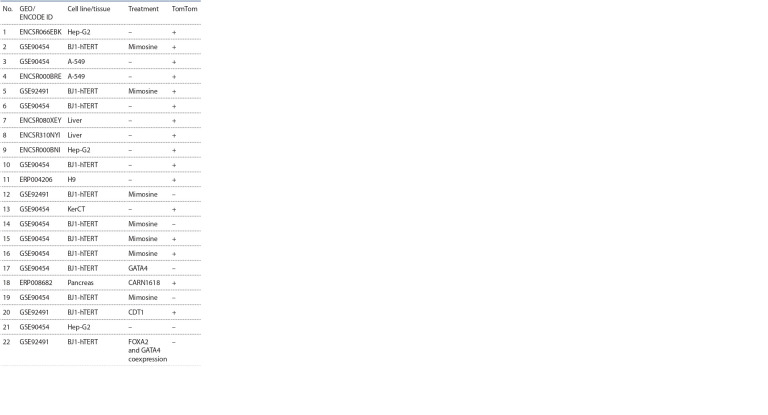
The list of ChIP-seq experiments used in our study Notе: GEO/ENCODE – unique identifier of databases (GSE*/ENC*). TomTom – result of filtering data using TomTom software (see “Comparison of found TFBS with known ones using TomTom tool”). (+)/(–) – the frequency matrix built on the basis of the TFBS found by ChIPMunk (PWM) is significantly similar (p-value<0.001)/not similar (p-value>0.001) to the frequency matrix of the FOXA2 TFBS from HOCOMOCO FOXA2_HUMAN.H11MO.0.A.

The input of our pipeline includes a dataset of ChIP-seq peaks with notation of genome version (mm10 or hg38) and the list of available TFBS search programs (PWM, diPWM, BaMM, InMoDe). The notation of genome version allows selection of the list of promoters in the fasta format (5′-regions of protein-coding genes, 2000 bp upstream transcription start sites). This promoter dataset is required for concordant threshold selection for all models. The total sizes of these samples were 19 795/19 991 genes for the human/ mouse genomes (GRCh38.p13/GRCm38.p6 versions). We used the reference genome to extract nucleotide sequences of the peaks

**Fig. 1.**
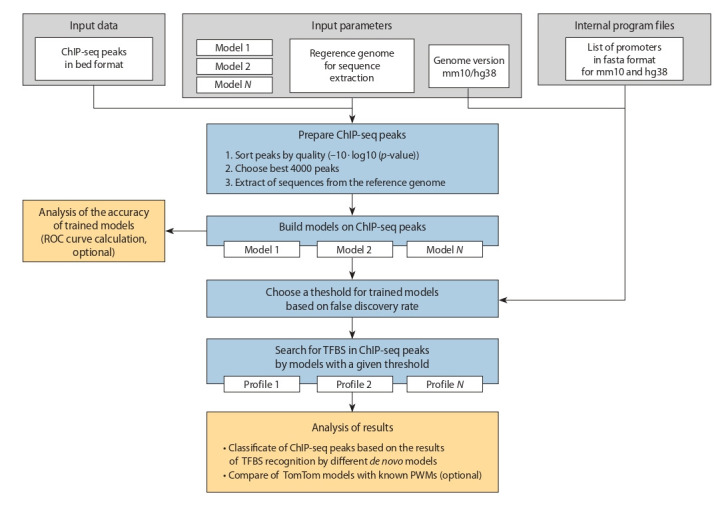
The scheme of MultiDeNA workflow.

**Pipeline for searching heterogeneity of TFBS.** We have developed the MultiDeNA pipeline (multiple de novo analysis, https://github.com/ubercomrade/MultiDeNA) to search TFBS in ChIP-seq data with several de novo models. This pipeline allows obtaining the classification of ChIPseq peaks, which is used to estimate the structural diversity of TFBS. The pipeline currently uses ChIPMunk (PWM), diChIPMunk (diPWM), BaMM, and InMoDe models, as well as the bedtools (Quinlan, Hall, 2010) and TomTom (Gupta et al., 2007) support programs. The schematic diagram of the program pipeline is shown in Fig. 1. The pipeline includes the following steps: (1) data preparation, (2) building of a model, (3) model accuracy assessment, (4) threshold selection and search of TFBS in ChIP-seq peaks with the fixed thresholds and (5) classification of ChIP-seq peaks according to results of TFBS recognition. Each stage of the program pipeline is described below

Preparing initial data for analysis. The preparation of the data included the sorting of peaks according the value –10∙log10 (p-value) that characterized the peak quality. This value was previously calculated for each peak by the MACS program (Zhang Y. et al., 2008). The pipeline of ReMap database (Chèneby et al., 2020) used this program to process raw ChIP-seq data. For each ChIP-seq dataset, we took in analysis top-scoring 4000 peaks. Next, nucleotide sequences of the peaks we extracted from the genome using bedtools (Quinlan, Hall, 2010).

Training de novo models and assessing the TFBS recognition accuracy. In order to recognize TFBS in peaks, it is necessary to build de novo models. The PWM and diPWM models we build with ChIPMunk and diChIPMunk, respectively (Kulakovskiy et al., 2010, 2013).The construction of alternative models we carry out with BaMM (Siebert, Söding, 2016) and InMoDe (Eggeling et al., 2017).

To improve the recognition accuracy of PWM model, we selected it optimal length by the cross-validation procedure. We used the same length for the construction of other models. This procedure included the following steps: (1) to divide the ChIP-seq dataset randomly into the training (90 % of the peaks) and the control (remaining 10 % of the peaks) samples; (2) to build a model with the training sample; (3) to get recognition scores of a model with the control sample to calculate true positive rate (TPR); (4) to generate the sample of random sequences by shuffling of nucleotides in the control sample; (5) to get scores of a model with the sample of random sequences to calculate the false positives rate (FPR); (6) repetition of steps 1–5 several times; (7) to calculate the ROC-curve (receiver operating characteristic). We compared different models with the pAUC value (partial area under curve), we calculated it as the part of the area under ROC curve for all FPR values less than 0.001 (McClish, 1989; Siebert, Söding, 2016). The method described above for choosing the optimal PWM length was developed earlier (Levitsky et al., 2007; Kulakovskiy et al., 2013). The accuracy of all models we assessed with the same approach

Next, a model can be applied to a nucleotide sequence with the same length as a model site. The result of applying this model is the recognition score. The larger score points to the higher probability of estimated nucleotide sequence to be a functional TFBS.

Threshold selection for models according to false positive rate estimates. To compare the results of TFBS search of different models correctly, it is necessary to set thresholds for all models uniformly. We set these thresholds for all models according the fixed FPR. To calculate this FPR, we use the negative sample, which included 5′-regions of protein-coding genes (2000 base pairs from transcription start sites).

**Fig. 2.**
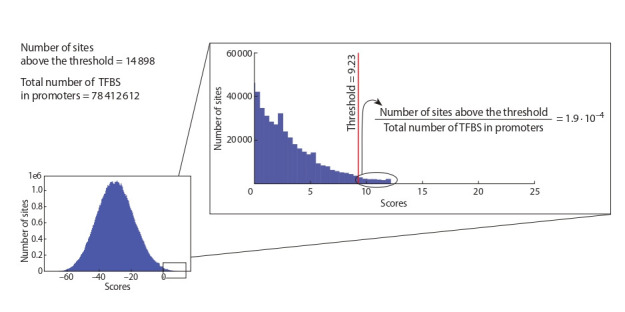
The approach of threshold selection for a model through estimation of false positive rate with the whole-genome promoter dataset.

We calculate FPR as follows. The scores of a model we determine for each site in the negative sample at each position and DNA strand. Then, the FPR for each unique score threshold we calculate as the ratio of the total count of predicted TFBS, for which the score is the same or higher than this threshold, to the total number of positions in the sequence sample available for such TFBS. We choose for recognition of TFBS in peaks thresholds for all models respecting the FPR 1.9 ∙10–4. An example of choosing a threshold for PWM for the GSE92491 dataset is shown in Fig. 2.

**Fig. 3.**
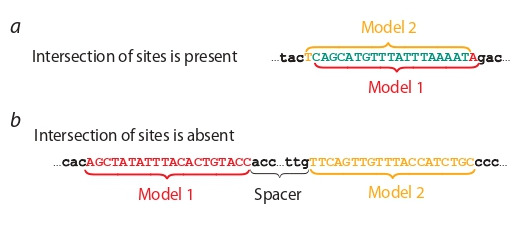
The example of classification for two ChIP-seq peaks containing sites of two various models (Model 1, Model 2). Colors mark options of intersected (a) or not intersected sites (b).

Classification of ChIP-seq peaks based on the results of TFBS recognition by different models. After threshold selection for all models, we search TFBS in ChIP-seq peaks. Further, these peaks we classify into fractions depending on the presence/absence of sites of different models (PWM, diPWM, BaMM, InMoDe). We use two types of classification. One of them take into account the location of TFBS of different models in a peak, and another did not (see previously developed method, Levitsky et al., 2014, 2016). In particular, we carry out for each pair of models the classification of peaks with taking into account positions of TFBS of different models. Totally, there are six pairs of models: PWM and diPWM, PWM and BaMM, PWM and InMoDe, BaMM and diPWM, BaMM and InMoDe, InMoDe and diPWM. If a peak includes TFBS of a single model only, then this peak we classify as the peak of the corresponding model. If there are only two different models with hits in a peak, then two outcomes are possible (Fig. 3).

**Fig. 4.**
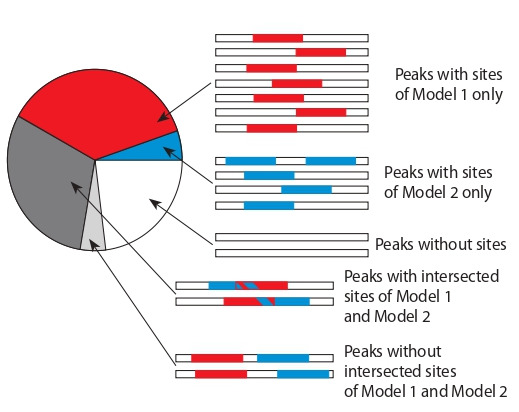
Peak classification for two models (Model 1, Model 2) with taking into account the intersection of TFBS.

In the first case, if there is at least one pair of sites from two models that has at least one common position, then such peak we classify as the “intersection”. Otherwise, if a peak contains sites of different models, but these sites are not intersected, then a peak is classified as “no intersection”. If sites are absent in a peak, then we classify it as “no sites”. Such classification of ChIP-seq peaks for the two models can be represented as the pie chart (Fig. 4)

The classification of peaks, without taking into account positions of sites of different models we carry out as follows. We identify following groups of peaks: peaks with sites of one model only, peaks containing sites of all models, and also several groups of peaks respecting combination of various models. 

**Comparison of found TFBS with known ones using TomTom tool.** To assess whether a predicted site matches to known FOXA2 sites, we use the TomTom motif comparison program (Gupta et al., 2007). This program is designed to assess the similarity between nucleotide frequency matrices. For each PWM model, we construct a nucleotide frequency matrix based on the sites it find. Next, using TomTom, we evaluate the similarity of this matrix to the frequency matrix of the FOXA2 from the HOCOMOCO database (ID HOCOMOCO FOXA2_ HUMAN.H11MO.0.A, Kulakovskiy et al., 2018). If the p-value of the matrix comparison is below 0.001, then a ChIP-seq dataset we consider as enriched with FOXA2 BS (see the Table). 

**Statistical data analysis.** Data analysis and visualization we perform in the Python 3.8 programming language in the Jupyter environment using the numpy, matplotlib, seaborn, and statannot packages. The distributions of peak fractions respecting to various models we compare with the Mann–Whitney U-test, corrected for multiple comparisons (Bonferroni approach).

## Results

Filtering data based on TomTom’s motif comparison

To ensure that the trained models find sites corresponding to known FOXA2 sites we apply the filter based on the TomTom program. For this, we build the frequency matrices respecting a trained model and we compare it with the known matrix of FOXA2 from the HOCOMOCO database. This procedure left only 16 ChIP-seq datasets out of total 22 (see the Table), therefore, these 16 sets we use in further analysis.

Classification of ChIP-seq peaks without taking into account the intersection of TFBS positions found by different de novo models

**Fig. 5.**
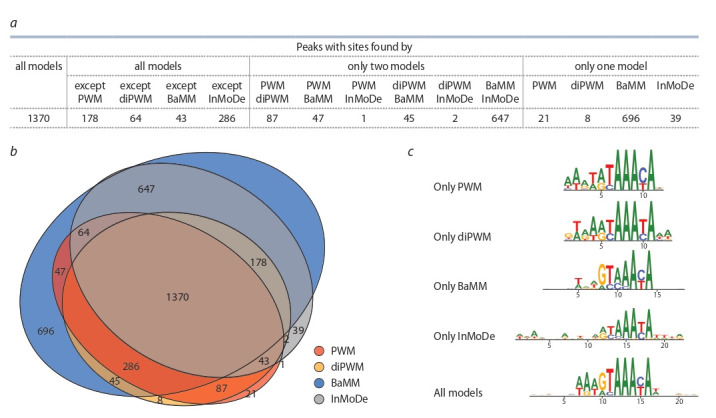
The classification of peaks of the GSE90454.FOXA2.KerCT ChIP-seq dataset according to prediction results of all four models. (a) Table, (b) Venn diagram, (c) Logo for fraction of the peaks respecting to predictions of sole models and that for the overlapping fraction of all models.

The main result of MultiDeNA pipeline is the classification of peaks. It allows establishing how the models are related to each other in terms of their ability to identify TFBS in peaks. We used two types of peak classification. The first one takes into account an intersection of positions of predicted TFBS of different models, the second one did not take it into account (see “Classification of ChIP-seq peaks based on the results of TFBS recognition by different models”). The example of results classification for GSE90454. FOXA2.KerCT dataset is given in Fig. 5.

Let us consider in more detail the classification of ChIPseq peaks based on the results of the TFBS search with four models without taking into account site positions. It can be seen that all models jointly recognized 88.35 % of the peaks (3534 out of 4000, the sum of all areas within the Venn diagram, see Fig. 5, a, b). The overlap fraction of all models amounts 34.25 % (1370 out of 4000 peaks). Two non-PWM models BaMM and InMoDe make the significant contributions to peak recognition. They totally add 34.55 % of all peaks (696+647+39 = 1382 out of 4000). This fraction is almost the same as the overlap fraction of all models (1370). The BaMM model makes the largest independent contribution to recognition of sites, it adds 17.4 % of the peaks (696), in contrast to other models that add 0.525 % (21), 0.975 % (39) and 0.2 % (8) (PWM, InMoDe and diPWM respectively).

To assess the structural diversity of the TFBS, we build logos for peak fractions “only PWM”, “only diPWM”, “only BaMM”, “only InMoDe” and “all models” (see Fig. 5, c). All logos contain the GTAAACA consensus. However, the “only PWM”, “only diPWM” and “only InMoDe” fractions have the higher occurrence of GT than AT at the first two nucleotides of the consensus. It can also be noted that the 5′-ends of all logos are diverse in nucleotide content.

Classification of ChIP-seq peaks with taking into account the intersection of TFBS positions found by different models

**Fig. 6.**
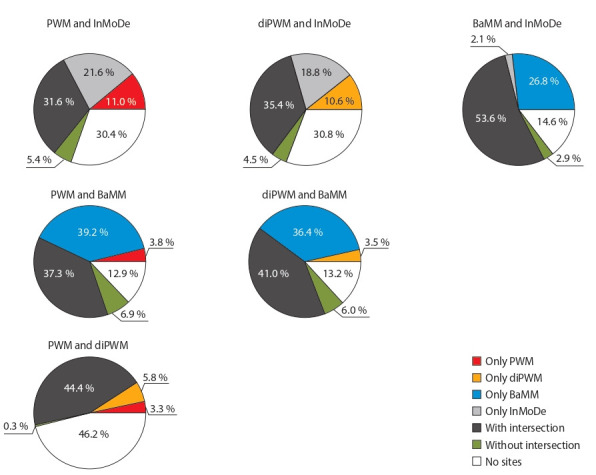
Classification of the GSE9045

The classification of peaks described above (without taking into account the positions of the TFBS) does not take into account positions of sites in peaks. To consider this circumstance we classify peaks with taking into account positions. We perform this for each pair of models (PWM– diPWM, PWM–BaMM, PWM–InMoDe, diPWM–BaMM, diPWM–InMoDe, InMoDe–BaMM). The results of the classification of peaks for GSE90454.FOXA2.KerCT are shown as the pie charts in Fig. 6

All pairs of model combinations have very small fraction of “without intersection” peaks, ranging from 0.3 to 6.9 %. On the other hand, all cases were characterized by the large fraction of peaks “with intersection” (BaMM– InMoDe 53.6 %, PWM–diPWM 44.4 %, diPWM–BaMM 41.0 %, PWM–BaMM 37.3 %, diPWM–InMoDe 35.4 %, PWM–InMoDe 31.6 %). This fraction is larger for methodologically close pairs of models BaMM–InMoDe and PWM–diPWM (see Fig. 6). The fraction of the peaks with TFBS found with only a single model is the highest for BaMM model. In pairs of models PWM–BaMM, diPWM– BaMM, and InMoDe–BaMM, BaMM contributes greatly (39.2, 36.4 and 26.8 %, respectively).

Evaluation of the recognition TFBS accuracy by different models for FOXA2 

**Fig. 7.**
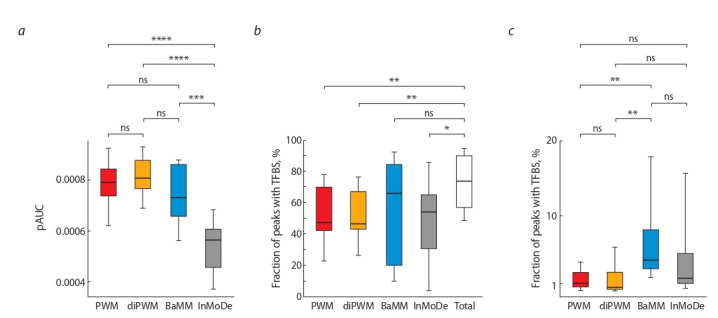
The distribution of quartiles for recognition measures. The bottom part of the box denotes the minimum value of parameter; the top part denotes the maximum value of parameter. (a) Values of pAUC for all models. (b) Fractions of peaks recognized with a single models (PWM, diPWM, BaMM, InMoDe) and with all models together (Total). (c) Fractions of peaks contained only TFBS recognized with a single model. ns – p > 0.05; * p < 0.05; ** p < 0.01; *** p < 0.001; **** p < 0.0001.

To compare the recognition accuracy of different models we calculate pAUC values from ROC curves obtained with the cross-validation procedure (see “Training de novo models and assessing the TFBS recognition accuracy”) (Fig. 7, a). According to the results obtained, the values of the pAUC medians for the PWM, diPWM, BaMM and InMoDe models are 8.0E–4, 8.1E–4, 7.3E–4, and 5.6E–4, respectively. The differences between pAUC values were not significant ( p > 0.05) for paired comparisons of PWM, diPWM, and BaMM, but the InMoDe model has significantly less values than any other model (p < 0.05).

Comparison of fractions of peaks with TFBS found by each model with that for all models. To investigate contributions of different models to the efficiency of TFBS search and to evaluate the overall result of several models, we determine fractions of peaks containing at least one TFBS for each sole model and those for all models together (see Fig. 7, b). The medians of recognized peaks fractions are 47.3, 46.4, 65.8, and 54 % for sole PWM, diPWM, BaMM and InMoDe, respectively. The median of recognized peaks fraction of joined results of all four models’ case is 73.6 %. Consequently, together, all models add 26.3 % peaks containing TFBS to the fraction of sole PWM model, which is consistent with the earlier obtained result of using two fundamentally different PWM and SiteGA models (Levitsky et al., 2014). At the same time, the median values respecting fractions for the PWM, diPWM, and InMoDe models significantly lower ( p < 0.05) than that obtained by combining all models. Thus, the approach using the combination of different models allows better identification of peaks with TFBS for FOXA2 than that using only one model. However, for BaMM, the fraction of recognized peaks did not statistically differ ( p > 0.05) from the result obtained by combining the four models. Hence, the BaMM model makes the main contribution to the recognition of FOXA2 peaks and, among the other models this model better describes the structure of FOXA2 sites. However, the rest three models add 7.8 % of the peaks to the BaMM result, which proves the effectiveness of using different models together.

Comparison of fractions of peaks containing TFBS found by single models. As it is shown above, the combination of different models increases the number of peaks with TFBS. Hence, each model recognizes TFBS that others do not. To assess the independent contributions of all models to the search for TFBS, we determine the fractions of peaks containing TFBS of only one model (see Fig. 7, c). As one can see, each model (PWM, diPWM, BaMM, InMoDe) is able to find TFBS that other models do not find. The medians of peaks containing TFBS respecting a single model are 1.08, 0.49, 4.15, and 1.73 %, respectively for PWM, diPWM, BaMM, and InMoDe. At the same time, the results for BaMM are significantly different ( p < 0.05) from those for both PWM and diPWM. It also confirms the assumption that the BaMM model better recognizes FOXA2 sites. However, each model contributes to site recognition. Consequently, each model reveals certain structural variant of TFBS.

Cross-validation test for PWM models with participation of their own training dataset and other ChIP-seq datasets 

**Fig. 8.**
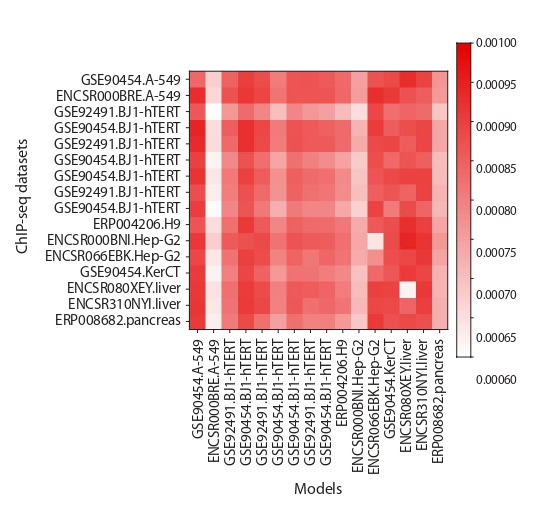
The heatmap of cross-validation test results for PWM models Colors mark pAUC values. Each diagonal cell implies that control and training datasets are the same. Remaining cells refer to distinct training and control datasets. Rows mean models and columns denote ChIP-seq datasets.

To estimate the dependence of specificity of various models for different ChIP-seq datasets as a function of a selection of particular dataset as the training sample, we performed the cross-validation test as follow. The accuracy of each PWM model we assessed not only within the same ChIPseq training dataset, but also for the rest 15 datasets (control datasets). For the case of training dataset, we performed several iterations to divide the total training sample into 90 % of the peaks that we used to build a model, and the remaining 10 % of the peaks we used to estimate the performance. For each case we calculated the accuracy estimate pAUC (see “Training de novo models and assessing the TFBS recognition accuracy”), the results we presented in the form of the heatmap (Fig. 8). The heatmap shows that only in three cases ENCSR000BRE.A-549, ENCSR000BNI.Hep-G2 and ERP008682.pancreas other models have very low pAUC scores, and for five cases GSE90454.A-549, ENCSR066EBK.Hep-G2, GSE90454. KerCT, ENCSR080XEY.liver and ENCSR310NYI.liver, all models have high pAUC values.

## Discussion

Based on all data obtained, we conclude that the joint use of alternative models allows us to expand the number of detected peaks containing TFBS relative to application of sole PWM.

This result can be explained by the presence of different structural types of TFBS of FOXA2. This is in agreement with experimental data obtained for a number of other TFs, including members of the FOX family. Thus, it was shown that HOXB13 and FOXC2 are able to bind with the same affinity to completely different sequences CAATAAA/ TCGTAAA (Morgunova et al., 2018) and GTAAACA/ ACAAATA (Chen et al., 2019), respectively. It was recently found that TF FOXN3 is able to bind to two fundamentally different types of TFBS, which had different lengths (Rogers et al., 2019). In addition, small changes in the structure of the TFBS depend on the cooperative interaction between TFs (Morgunova, Taipale, 2017). Hence, we propose that FOXA2 also can bind to different structural types of BS.

To take into account all the TFBS structural types, the only PWM model for site recognition may not be enough, this problem partially was solved using several PWMs (Bi et al., 2011; Mitra et al., 2018) or using alternative models (Mathelier, Wasserman, 2013; Yang et al., 2014; Siebert, Söding, 2016; Eggeling et al., 2017; Gheorghe et al., 2019). However, previously alternative models were usually compared with PWM only in terms of the recognition accuracy (Siebert, Söding, 2016), or according the number of recognized TFBSs (Samee et al., 2019). In the current study, we took in analysis FOXA2 ChIP-seq data. We compared not only the accuracy and the number of peaks recognized, but also we estimated independent contributions of each model and assessed the joint contribution for all pairs of models, and also we tested positions of hits in peaks for each pair of models. The results for the accuracy assessment (see Fig. 7, a) showed that the InMoDe model had the lowest accuracy relative to other models, and the BaMM, diPWM and PWM models were comparable in accuracy. In terms of expanding the total fraction of peaks with TFBS, the BaMM model performed the best, since this model found the largest fraction of peaks with TFBS that other models do not find. Nevertheless, all alternative diPWM, BaMM and InMoDe models allow expanding the pool of recognized TFBS relative to sole PWM, but PWM also makes an independent contribution to the total number of peaks with recognized TFBS.

## Conclusion

We have developed the pipeline MultiDeNA, which allows uniform processing of ChIP-seq data using different TFBS models. Currently, it can be used to build PWM, diPWM, InMoDe, BaMM models. MultiDeNA includes the steps of preparing data, building models, evaluating recognition accuracy, scanning peaks, combining results, and analyzing them. The developed pipeline of programs processed datasets from the ReMap database, including 22 ChIP-seq experiments for TF FOXA2. We have shown that combined use of different models allows increasing the total fraction of recognized peaks up to 73.6 % (relative to sole PWM model, the fraction of recognized peaks increased by 26.3 %). We have shown that different models tend to recognize the same sites of FOXA2 in the large fraction of peaks, thereby revealing the most common structural type of TFBS in these peaks. Also, each model found TFBS that other models did not predict. The BaMM model performed the best with 4.15 % of peaks containing only its sites, versus 1.08, 0.49, 1.73 % for PWM, diPWM and InMoDe, respectively. We proposed that the heterogeneity of sites for FOXA2 is revealed only if two or more models are applied. The diPWM model showed worst result in sole application in comparison with other models (diPWM recognized TFBS in 46.4 % of the peaks). The best model for the FOXA2 sites was BaMM; it found TFBS in 65.8 % of the peaks. Hence, we assumed that the BaMM model could better describe BS for FOXA2.

## Conflict of interest

The authors declare no conflict of interest.
